# Über Geschmack lässt sich nicht streiten? Zum epistemologischen Status der Geschmackswahrnehmung in der speziellen Pharmakologie Galens

**DOI:** 10.1007/s00048-023-00358-x

**Published:** 2023-05-15

**Authors:** Maximilian Haars

**Affiliations:** Universität Marburg, Marburg, Deutschland

**Keywords:** Galen von Pergamon, Antike Pharmakologie, Pharmakognosie, Sinnliche Wahrnehmung, Geschmacksqualitäten, Galen of Pergamon, Ancient Pharmacology, Pharmacognosy, Sensory Perception, Taste Qualities

## Abstract

In diesem Artikel wird eingehend untersucht, welche auf den Erwerb von Wissen bezogene Rolle die Geschmackswahrnehmung in der Forschung zu den einzelnen Pharmaka bei dem römischen Arzt Galen von Pergamon spielt. Hierzu wird 1.) durch eine Quellenuntersuchung plausibel gemacht, dass die gegenüber seinen Vorgängern – insbesondere Dioskurides und Sextius Niger – mitunter vermehrten, detaillierteren und abweichenden Geschmacksangaben auf eigenen Forschungen Galens beruhen, 2.) die Forschungspraxis Galens rekonstruiert und 3.) die Darstellung seiner Ergebnisse in sprachlicher und logischer Hinsicht untersucht sowie die Besonderheit gegenüber der traditionellen Arzneimittellehre herausgestellt. Ausgehend davon wird dafür plädiert, a) dass der gustatorischen Wahrnehmung bei Galen eine besondere Bedeutung zukommt, die über ihre traditionell deskriptive Funktion weit hinausweist, indem er b) ausgehend von der Erkenntnis, dass Geschmack und Arzneimittelwirkung zusammenhängen, den Geschmacksqualitäten eine Indikatorfunktion zuweist für ein viel grundlegenderes, die Arzneimittelwirkung verursachendes Prinzip, und somit c) den Boden bereitet hat für eine auch nach Geschmacksprinzipien einteilende Pharmakognosie, die ihren Höhepunkt erst sehr viel später erreichen sollte. Mit Blick auf eine bis heute ausgeübte Praxis der gustatorischen Prüfung pflanzlicher Drogen in der Pharmazie, die wie jede andere naturwissenschaftliche Disziplin den sinnlichen Bezug zu ihrem Gegenstand ansonsten weitgehend verloren hat, möchte der Beitrag daher einen Anreiz geben, die „Geschichte pharmazeutisch-medizinischen Schmeckens“ zu studieren.

## Abstract (en)

This article examines the role of taste perception in Galen’s research on simple drugs in relation to the acquisition of knowledge. To this end, 1.) I make it plausible through an examination of sources that the sometimes increased, more detailed and divergent indications of taste compared to his predecessors, especially Dioscorides and Sextius Niger, are based on Galen’s own research, 2.) reconstruct Galen’s research practice and 3.) examine the presentation of his results in linguistic and logical terms and explain the differences to traditional pharmacology. I argue that a) gustatory perception has a special significance in Galen’s work that goes far beyond its traditional descriptive function and b) that, starting from the realisation that taste and drug action are interrelated, he assigns to taste qualities an indicator function for a much more fundamental principle that causes drug action and thus c) has prepared the ground for a pharmacognosy that also classifies according to taste principles, which was to reach its climax much later. With a view to a practice of gustatory testing of herbal drugs that is still practised today in pharmacy, which, like any other natural science discipline, has otherwise largely lost the sensory reference to its subject matter, the article would therefore like to provide an encouragement to study the “history of pharmaceutical-medical tasting”.

Vor 50 Jahren veröffentlichte Georg Harig in ebendieser Zeitschrift einen Artikel, der einem zentralen – wenn nicht gar dem wichtigsten – Aspekt in der theoretischen Pharmakologie des römischen Arztes Galen von Pergamon (129–ca. 216 n. Chr.) gewidmet war: dem Verhältnis zwischen Primär- und Sekundärqualitäten bei der Beurteilung der aus den drei Naturreichen gewonnenen Pharmaka (Harig 1973).

Das maßgeblich von Galen begründete System hat im weiteren Verlauf der europäischen und orientalischen Medizingeschichte eine derart breite Rezeption erfahren, dass es bis in die Frühe Neuzeit hinein kaum ein Werk zur Arzneimittellehre gibt, das auf die Einteilung der Drogen in „warme“, „kalte“, „trockene“ und „feuchte“ verzichten würde. Für die Ermittlung dieser sogenannten Primär- oder Elementarqualitäten ist aber, wie Galen ausführlich begründet und Harig genau herausgearbeitet hat, die Feststellung der Sekundär-, und zwar insbesondere der Geschmacksqualitäten gefordert. Der gustatorischen Wahrnehmung kommt folglich in der Pharmakologie Galens eine besondere Bedeutung zu, die über ihre traditionelle Funktion in der deskriptiven pharmakognostischen Literatur weit hinausgeht.

In diesem Beitrag soll es indessen nicht um ihre theoretische Fundierung gehen (die, nebenbei bemerkt, nicht nur durch die Arbeiten Harigs bereits die Aufmerksamkeit der wissenschaftshistorischen Forschung auf sich gezogen hat)^1^, als vielmehr darum, „was Wissenschaftler tun, wenn sie ihre jeweilige Forschung betreiben“ (Rheinberger 2013: 12) – also um ihre praktische Anwendung in der Untersuchung der einzelnen Pharmaka in der speziellen Pharmakologie. Um schließlich nachvollziehen zu können, welche Rolle ihr beim Erkenntnisgewinn tatsächlich zukam, wird unsere Untersuchung die jeweiligen Angaben Galens auch vor dem Hintergrund seiner pharmakologischen Vorgänger in ihrer historischen Entwicklung in den Blick zu nehmen haben und damit – hier ist auf das Wohlwollen des Lesers zu hoffen – auch besonders nach ihren Quellen schmecken.

## Bittere Wurzeln – süße Früchte: Geschmacksangaben in der Pharmakognosie

Wer heutzutage in Deutschland ein Studium der Pharmazie aufnimmt, durchläuft im Fach Pharmazeutische Biologie auch einen atavistisch anmutenden Kursus, der sich unter den Student*innen allerdings allgemeiner Beliebtheit erfreut: Es geht darum, über 100 verschiedene Arten von Wurzeln, Blättern, Kräutern, Früchten und so weiter mit allen Sinnen wahrzunehmen, auch – oder gerade – ihren Geschmack zu prüfen und die Eindrücke genau zu protokollieren, damit diese sogenannten „Drogen“ daraufhin einwandfrei identifiziert werden können.

Es bedarf kaum einer historischen Vorbildung für die Einsicht, dass diese Art der wissenschaftlichen Praxis ihre Wurzeln in einer Zeit hat, zu der instrumentelle Analytik noch nicht zur Verfügung stand und der Heilkundige alle Möglichkeiten organoleptischer Prüfung auszuschöpfen hatte, um die Qualität einer Droge zu bestimmen. Manche mögen heute zweifeln, wie ein Geschmacksurteil, über das sich laut einem Sprichwort nicht streiten lässt (was bedeutet, dass die Subjektivität des Urteils jedem zugestanden wird), zu objektivierbaren Ergebnissen führen soll. Doch ein solcher Zweifel wäre antiken Ärzten wie Galen nicht nur fremd (Singer & van der Eijk 2018: 19), sondern ist auch, wie ich hoffe zeigen zu können, in Bezug auf die Pharmakognosie weitgehend unbegründet.

Galen entfaltet seine Arzneimittellehre in mehreren Schriften, wobei die in ihrer letzten Edition knapp 900 Seiten zählende Abhandlung *Über die Wirkungspotenziale der einfachen Arzneimittel*^2^ systematisch gesehen den ersten Platz einnimmt. Die Schrift gliedert sich ähnlich wie moderne Lehrbücher der Pharmakologie in eine einleitende allgemeine (Bücher I–V) und eine spezielle (VI–XI) Arzneimittellehre – ein Katalog mit den von Dioskurides beschriebenen Vegetabilia, Mineralia und Animalia. Im letzten Buch (XI) dieser Schrift kommt Galen bei der Beurteilung von Fetten und Talgen auch auf seine Vorgänger zu sprechen. Durch längere Lagerung komme es zur Qualitätsveränderung; die Fette würden im Geschmack „beißender“ (δριμύτερα), nicht jedoch „adstringierender“, was einige Autoren zu Galens Missfallen behauptet haben (XII 329.9–17 K.):

Einige von denjenigen [medizinischen Autoren], die die Bedeutungen der Wörter korrumpieren (τινὲς δὲ τῶν διαφθειρόντων τὰ σημαινόμενα τῶν ὀνομάτων), nennen alle derartigen [Stoffe] nicht „beißend“ (δριμέα), sondern „adstringierend“ (στύφοντα), bis hin zum Pfeffer – als ob es keinen Unterschied machte, entweder „adstringierend“ oder „beißend“ zu sagen. Und wenn man sie wiederum nach Galläpfeln, Myrten, Mispeln, Granatapfelschalen, unreifen Oliven und Gerbersumach fragen würde, dann behaupten sie, dass auch diese adstringieren, obgleich wir ja hierbei die gegensätzlichste Wahrnehmung haben zu derjenigen, die uns durch Pfeffer, Feuerwurzel-Bertram und Senf […] entsteht.^3^

Zu den die Wortbedeutung korrumpierenden Autoren zählt, wie Galen im Folgenden (XII 350.12 K.) deutlich macht, insbesondere der Anazarbeer Dioskurides – also seine pharmakologische Hauptquelle. Man kann sich sicherlich fragen, ob es nicht Pedanterie, vielleicht auch Polemik oder die von diesem Autor bekannte „Gier nach ausschließlicher Leserbindung“ in einem „Agon aller gegen alle“ (Asper 2005: 33) ist, über die Geschmacksurteile längst verstorbener Ärzte zu streiten. Damit würde man allerdings ein wesentliches Charakteristikum von Galens Pharmakologie (und auch denjenigen der Folgezeit) verkennen. Für Galen war die Sinneswahrnehmung zunächst ein valides und notwendiges Mittel, das in der medizinischen Forschung stets hinzuzuziehen und an dem die theoretischen Überlegungen jederzeit zu prüfen sind. Genau genommen macht Galen auch nicht den Fehler, Dioskurides absprechen zu wollen, einen bestimmten Geschmackseindruck gehabt zu haben (denn darüber lässt sich wirklich nicht streiten), sondern er kritisiert nur die Nachlässigkeit des Sprachgebrauchs bei einem Autor, der als Nicht-Muttersprachler gar in seinem Vorwort den Leser bittet, das Augenmerk nicht auf seine Ausdrucksfähigkeit zu legen (μὴ τὴν ἐν λόγοις δύναμιν ἡμῶν σκοπεῖν, Dsc. Prooem 5). Um eine entsprechend exakte Terminologie, die insbesondere auch den Sprachgebrauch seiner eigenen Zeit berücksichtigt, war Galen folglich äußerst bemüht.

Das gesamte vierte und fünfte Buch seiner Schrift (XI 619–788 K.) ist im Wesentlichen mit der Frage beschäftigt, wie die Mischung (*krasis*) der Pharmaka mit ihren wärmenden, kühlenden, trocknenden und feuchtmachenden Eigenschaften (*dynameis*) einerseits und den Geschmacksqualitäten (Buch IV) sowie den spezifischen Arzneimittelwirkungen (Buch V) andererseits zusammenhängen. Prinzipiell gehen seine Überlegungen von den Prämissen aus, dass sowohl die Geschmacksqualitäten als auch die Arzneimittelwirkungen ihre Ursache in einer bestimmten *krasis* der Elementarqualitäten haben und dass ferner zunächst „die Geschmacksqualität eines Pharmakons die Äußerungsform seiner medizinischen Wirkung im oder am Körper darstellt“ (Harig 1973: 69). Entsprechend ausgearbeitet ist seine Geschmackslehre, die in der Tradition Platos (*Timaios* 28: 65c–66c) und des Peripatos (Theophrast *De caus. pl.* VI 4.1) steht und insgesamt neun Qualitäten unterscheidet: 1. adstringierend (στῦφον/*styphon*, 136×), 2. herb-sauer (στρυφνόν/*stryphnon*, 27×), 3. herb (αὐστηρόν/*austēron*, 15×), 4. stechend-sauer (ὀξύ/*oxy*, 7×), 5. süß (γλυκύ/*glyky*, 18×), 6. fettig (λιπαρόν/*liparon*, 3×), 7. salzig (ἁλυκόν/*halykon* oder ἁλμυρόν/*halmyron*, 4×), 8. bitter (πικρόν/*pikron*, 114×) und 9. beißend(-scharf, δριμύ/*drimy*, 110×).^4^ Der Geschmackssinn erhält bei Galen insofern seine besondere Bedeutung, als er vor allen anderen Sinnen geeignet ist, Hinweise zur *dynamis* eines Arzneimittels zu liefern. Die von Harig genauer untersuchte Ableitung der Geschmacksqualitäten kann an dieser Stelle nur gerafft wiedergegeben werden: Die adstringierenden, das heißt „zusammenziehenden“ Qualitäten – man denke beispielsweise an einen gerbstoffreichen Wein (1.–3.) – werden von der kalten Primärqualität abgeleitet, die bei (3.) allerdings weniger stark ausgeprägt sei. Geschmack (4.) ist ebenfalls kalt, allerdings von besonders „feinteiliger Substanz“. Die typischen Geschmacksqualitäten der Nahrungsmittel (5.) süß und (6.) fettig würden in ihrer Temperierung der Wärme des menschlichen Körpers entsprechen und daher als angenehm empfunden. Die übrigen drei Qualitäten seien entsprechend Ausdruck der warmen Elementarqualität, an der sie einen graduell ansteigenden Anteil haben, wobei die äußerst warme, scharfe Qualität (9.) aufgrund ihrer gleichzeitigen „Feinteiligkeit“ besonders beißend sei (man denke an Pfeffer, Zwiebeln und Knoblauch).^5^ In der obigen Aufzählung ist ferner die Anzahl an Vegetabilia angegeben, denen bei Galen die jeweilige Qualität zugeschrieben wird (nach Haars 2016: 194). Es zeigt sich schon hier: Medizin muss bitter schmecken!

## Geschmacksqualitäten in der Drogenkunde vor Galen

Um beurteilen zu können, in welchem Umfang die Geschmacksangaben in der speziellen Pharmakologie Galens (*De simpl. med.* Bücher VI–XI) auf eigenen Forschungen basieren oder aber auf seine Vorgänger zurückzuführen sind, sollen im Folgenden insbesondere die Autoren betrachtet werden, die Galen heranzog. Da diese Autoren unsere Hauptquellen zur antiken Arzneimittellehre darstellen, dürfte die folgende Untersuchung auch insgesamt repräsentativ für diesen Zweig der griechischen Heilkunde sein.

Die zu Galens Zeit und weit darüber hinaus berühmteste und ausführlichste Arzneimittellehre war diejenige des Anazarbeers Dioskurides aus der zweiten Hälfte des ersten nachchristlichen Jahrhunderts. Galen (XI 794 K.) kannte sie gut und lobt sie ausdrücklich, wie auch ein ähnliches, in der ersten Hälfte desselben Jahrhunderts entstandenes griechisches Werk des Sextius Niger (Wellmann 1889, Scarborough 2008), das als gemeinsame Quelle sowohl bei Dioskurides als auch bei Plinius eingeflossen ist (im Folgenden abgekürzt: e S. N., nach Wellmann 1907–1914). Aus den Übereinstimmungen beider Autoren kann man seine Lehre herausschälen, weswegen wir im Folgenden auch Plinius betrachten, obwohl Galen ihn nicht erwähnt – wie überhaupt keinen ausschließlich in lateinischer Sprache schreibenden Autor (vgl. Nutton 2013: 395, Anm. 89).

Hierzu wurde das dritte Buch von *De materia medica* (hg. Wellmann 1907) ausgewertet, worin unter anderem Heilpflanzen der wichtigen Familien Apiaceae, Asteraceae und Lamiaceae behandelt sind. Um den deskriptiven Charakter dieser Angaben zu zeigen, müssen einige Beispiele genügen: Von den 158 Kapiteln, in denen meist eine Arzneipflanze (zuweilen auch mehrere ähnliche Arten) abgehandelt wird, finden sich in 78 – also fast der Hälfte – Geschmacksangaben. Stichproben der anderen Bücher zeigen Ähnliches: In 129 Kapiteln des ersten und 192 des vierten Buches finden sich Angaben zu 81 (hier werden viele aromatische Pflanzen und Obstarten behandelt) beziehungsweise 79 Pflanzen (sie fehlen bei vielen giftigen Arten). Die häufigsten Geschmacksqualitäten der *Simplicia* sind beißend-scharf (δριμύς/*drimys*, 29×), brennend (πυρωτικός/*pyrōtikos*, 7×), bitter (πικρός/*pikros*, 21×), süß (γλυκύς/*glykys*, 8×), adstringierend (Formen von στύφειν/*styphein*, 6×), sauer (στρυφνός/*stryphnos*, 2×), salzig (ὕφαλμος/*hyphalimos*, 1×) und aromatisch (Formen von ἀρωματίζειν/*arōmatizein*, 6×).

Ein genauerer Vergleich des dritten Buches mit Plinius ergibt ferner, dass Dioskurides bezüglich der Geschmacksangaben fast immer ausführlicher ist. Dies überrascht nicht, denn man würde von vornherein annehmen, dass ein drogenkundliches Werk, das für Fachkreise bestimmt ist, eher auf die Geschmacksprüfung zur Identitätsfeststellung eingeht als eine naturkundliche Enzyklopädie für eine breitere Leserschaft. Zu den Angaben in 34 Kapiteln bei Dioskurides findet man etwa in den Parallelstellen bei Plinius überhaupt keine Entsprechung.^6^ So handelt Dsc. III 48 ausführlich die *Panazee des Herakles* ab, mit ihren „wohlriechenden, brennend-scharfen Samen“ und „Wurzeln, die eine dicke, im Geschmack leicht bittere Rinde besitzen“, von welchen diejenigen besser seien, die „im Geschmack brennend und aromatisch sind“, während sich unter den davon gewonnenen „Gummiharzen dasjenige mit dem bitteren Geschmack auszeichne“.^7^ Diese Angaben fehlen hingegen bei Plinius XII 127 (e S. N.), wie auch, dass der Samen von *ami *im Geschmack „origanisierend“ sei (so die Wortneubildung von Dsc. III 62: ὀριγανίζον τῇ γεύσει – fehlt bei Pl. XX 163f. e S. N.). Auch die Geschmacksbeschreibung der berühmten Droge *Silphium* bietet nur Dsc. III 80: „Es zeichnet sich derjenige Saft aus, der weder lauchartig noch unangenehm im Geschmack ist [… D]er Kyrenäische Saft […] ist auch im Geruch so ausgesprochen milde, dass auch nach dem Kosten kein Mundgeruch entsteht“.^8^ In zwei Fällen erwähnt Plinius die Heilpflanze nicht.^9^

In 25 anderen Fällen stimmen diese bei beiden Autoren genau überein (wenn man eine gewisse Toleranz durch die Übersetzung der griechischen Quelle ins Lateinische bei Plinius zulässt).^10^ So sagt Dsc. III 1 von dem Baumpilz *agarikon*, dass er zu Beginn süß schmeckt, beim weiteren Kauen aber bitter (γεύσει δὲ […] κατ’ ἀρχὰς μὲν γλυκάζοντα, εἶτα ἐξ ἀναδόσεως ἔμπικρα), was wir ebenso bei Plinius lesen (*initio gustus dulcis mox in amaritudinem transit*, Pl. XXV 103, e S. N.). Auf dieses Urteil werden wir unten bei Galen noch zurückkommen. Gerade da, wo der Geschmackstest wichtig ist, um Drogen zu unterscheiden, stimmen beide überein: Das Kraut Kretischer Diktam nennen sie (nach meinem Empfinden zu Recht) „beißend“ (Dsc. III 32: πόα […] δριμεῖα λίαν – *acre gustu*, Pl. XXV 92), und zwar schon in einer sehr geringen Menge (*minima portione accendit os*, PI. XXV 93^11^), während die Verfälschung, das Pseudodiktamnon, weniger beißend sei (Dsc. III 32.2: ἧττον δριμύ). Beide unterscheiden ferner die wilde von der kultivierten Raute anhand ihres beißenderen Geschmacks (Dsc. III 45: τὸ ὄρειον καὶ ἄγριον τοῦ ἡμέρου δριμύτερον – *ruta* […] *Silvestris* […] *ad omnia acrior*, Pl. XX 131 e S. N.). Weitgehende Einigkeit herrscht auch bei der Beurteilung der berühmten Droge Euphorbium, deren Geschmacksprüfung die antiken Autoren jedoch vor eine große Herausforderung stellte – vgl. Dsc. III 82: „Wähle aber den scharfen [Saft] aus. Allerdings ist er schwer mit dem Geschmackssinn nach der Einnahme zu beurteilen, denn wenn die Zunge einmal gebissen wurde, bleibt der brennende Geschmack eine ganze Weile, sodass alles [ihr] Zugeführte Euphorbium zu sein scheint“.^12^ Plinius, der mit seiner Quelle fälschlich den aus der Pflanze *chamelaia* bereiteten Saft für Euphorbium hält, sagt: „[E]r brennt, wenn auch nur leicht gekostet, lange im Mund und nach einer Weile noch heftiger.“^13^ Die nach Circe benannte Pflanze hat Wurzeln, welche Dsc. III 119 als „wohlriechend und wärmend“ beschreibt (κιρκαία […] ῥίζας […] εὐώδεις, θερμαντικάς). Dass „wärmend“ hier als Geschmack gemeint ist, bezeugt Plinius: *Circaea* […] *radice* […] *odorata, gustus calidi*, Pl. XXVII 60 (e S. N.).

In elf Fällen bieten beide Autoren voneinander abweichende Geschmacksangaben und in 80 Kapiteln fehlen Geschmacksangaben bei beiden, wobei die Gründe hierfür unklar sind.^14^ In sieben Fällen,^15^ wo bei Dioskurides Angaben fehlen, findet man solche bei Plinius, und zwar jeweils in Abschnitten, die aus Sextius Niger stammen. Was lernen wir daraus? Dass Dioskurides und Plinius ihrer gemeinsamen Quelle, dem römischen Arzt Sextius, auch in Hinblick auf die Geschmacksqualitäten viel verdanken, steht außer Frage. Unklar ist allerdings der Umfang, in dem Dioskurides ihn für Informationen herangezogen hat, die bei Plinius fehlen. Hier muss man davon ausgehen, dass der Anazarbeer eine andere Quelle hatte oder eigene Beobachtungen oder Geschmäcke darlegt. So bemerkt Dsc. III 54 über den Samen von Tordylon, dass er „leicht beißend und aromatisch sei“ (σπερμάτιον […] ὑπόδριμυ, ἀρωματίζον) – eine Angabe, die bei Plinius (XXIV 177 e S. N.) fehlt; ja er schreibt gar, dass er über die Pflanze nichts anderes in Erfahrung bringen (und das heißt wohl: bei Sextius lesen) konnte, als was er hierzu berichtet (*neque aliud de ea proditum invenio quam* […]). Dies ist freilich nur ein winziges, kaum belastbares Indiz, und da die Schrift des Sextius nicht überliefert ist, wird eindeutige Klarheit nicht zu gewinnen sein.

**Figure 1:**
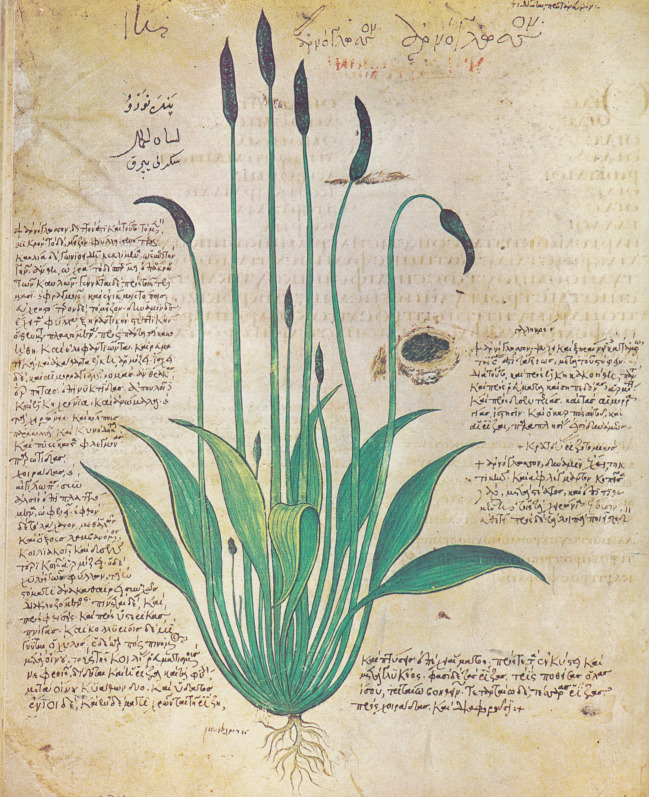

Durch die Quellenuntersuchung von Wellmann (1897) haben wir Anlass zu glauben, dass die pharmakognostischen Angaben des Sextius Niger unter anderem auf Krateuas, den Leibarzt von Mithridates VI. (ca. 132–63 v. Chr.), zurückgehen. Er verfasste nach den wenigen Zeugnissen, die überliefert wurden, ein bebildertes, alphabetisch geordnetes Kräuterbuch, das vielleicht als Vorbild des Wiener Dioskurides (Österreichische Nationalbibliothek, Cod. med. gr. 1 [Abb. 1]) gedient haben könnte (hierfür gibt es jedoch keinen Beweis, siehe auch Cronier 2009), ferner ein als „Wurzelschneiderbuch“ (*rhizotomikon*) betiteltes Werk mit Pflanzenbeschreibungen (aber ohne Bilder) und schließlich eine Schrift, in der auch mineralische Drogen behandelt wurden und die vielleicht Teil eines größeren Werks nach Art der dioskurideischen *De materia medica* gewesen ist (Wellmann 1897; Jacques 2008). Aus dem ersten Werk sind zehn Fragmente in dem Wiener Prachtcodex erhalten und ediert (bei Wellmann 1914: 144–146). Sie haben jeweils eine Arzneipflanze mit ihren Indikationen zum Gegenstand, wobei im Unterschied zu den korrespondierenden Kapiteln bei Dioskurides keine Geschmacksqualitäten genannt werden.^16^ Dies mag uns nicht überraschen, denn die Geschmacksqualitäten waren (bei Dioskurides und wohl auch vor ihm) Bestandteil des deskriptiven botanischen Teils, der ja in einem bebilderten Kräuterbuch entbehrlich war. Eine größere, wenn auch immer noch sehr dürftige Textmenge stellen die überlieferten Zeugnisse zu seiner Lehre dar (bei Wellmann 1914: 139–144). In diesen Testimonien, die fast ausschließlich die Synonyme der Pflanzen betreffen, finden sich nur wenige ausführlichere Zitate, die auch die Beschreibung der Gewächse, niemals aber ihren Geschmack zum Gegenstand haben. Eine Ausnahme ist allein die Stelle aus der pseudogalenischen Schrift „Über die Heilkräfte der Centaurea“, welche Krateuas im dritten Buch seines Werks behandelt haben soll (test. A 20). Wellmann hat in diesem Testimonium zudem die ganze Beschreibung der Centaurea mitsamt der Angabe „auch ist ihre Wurzel im Geschmack süß und leicht beißend“ (*et est* [sc. radix centaureae] *in gustu dulcis et subacris*) ausgehoben und legt damit nahe, dass hier Krateuas paraphrasiert wird – was wir freilich nicht wissen können. Zumindest sehe ich aber keine Gründe, die dagegensprächen (vgl. auch Nutton 2014: 152). Abschließend lässt sich festhalten, dass das überlieferte Krateuas-Material kein Urteil in der Frage zulässt, ob der Autor Geschmacksangaben systematisch erhoben hat oder nicht.

Ähnliches gilt auch für Diokles von Karystos (4. Jh. v. Chr.), obgleich wir hier deutlich mehr überlieferten Text besitzen. In dessen zuletzt von Philip van der Eijk (2000–2001) gesammelten Fragmenten begegnen uns gelegentlich Geschmacksqualitäten, meist jedoch in diätetischen Zusammenhängen (fr. 183a.104.119f.125, fr. 187.11–13 usw.). Diokles empfiehlt beispielsweise (das schon oben genannte) „Silphium, das am besten riecht und am bittersten [schmeckt]“ (σίλφιον δέ […] τὸ εὐωδέστερον καὶ πικρότατον, fr. 187.24f.). Aus den Übereinstimmungen von Pflanzensynonyma und -beschreibungen zwischen Dioskurides, Nikander und Theophrasts *Historia plantarum* (insbesondere Buch IX)^17^ schloss Wellmann (1898), dass Diokles’ *rhizotomikon* das älteste Kräuterbuch der Griechen darstellt. Diesem Wurzelschneiderbuch, das in unserem Zusammenhang am interessantesten wäre, kann jedoch nur ein einziges Fragment (fr. 204, ohne Geschmacksangabe) sicher zugeordnet werden. Bekannt ist Diokles ferner für das von Galen (VI 455f. K.) überlieferte sogenannte „große Methodenfragment“ (fr. 176), in welchem davor gewarnt wird, von Substanzen (im Kontext bei Galen: Lebensmitteln) ähnlichen Geschmacks notwendigerweise auf ähnliche (Nähr‑)Kräfte zu schließen („Diejenigen, die annehmen, dass Substanzen mit ähnlichem Geschmack […] alle auch die gleichen Kräfte besitzen, glauben dies zu Unrecht“; οἱ μὲν οὖν ὑπολαμβάνοντες τὰ τοὺς ὁμοίους ἔχοντα χυλοὺς […] πάντα τὰς αὐτὰς ἔχειν δυνάμεις οὐ καλῶς οἴονται) – eine Warnung, die sich insbesondere gegen undifferenzierte Generalisierungen richtet, nicht gegen Ursachenforschung überhaupt (van der Eijk 2000–2001: 331f.).

## Ein umfangreiches Forschungsprogramm: die Kartei geschmeckter Simplicia

Bevor wir uns Galen zuwenden, wollen wir kurz die Ergebnisse der vorangegangenen Untersuchung festhalten: Geschmacksqualitäten hatten bei den Autoren, die Galen heranzog, primär die Funktion, die Droge zu charakterisieren, ihre Qualität zu bestimmen, sie von anderen ähnlichen Sorten abzugrenzen oder Fälschungen zu erkennen. Die meisten Geschmacksangaben finden wir bei Dioskurides, nämlich zu durchschnittlich 50 Prozent der behandelten *Simplicia*. Zumindest ein großer Teil dieser Angaben findet sich auch bereits bei seiner wichtigsten Quelle, Sextius Niger. Dass auch dessen Quellen – nämlich Krateuas und möglicherweise Diokles – in größerem Umfang Geschmacksangaben erfasst oder weitertradiert haben, lässt sich zwar nicht mehr oder nur noch anhand weniger Fragmente für einzelne Beispiele zeigen, doch dürfte nach dem Charakter dieser Literaturgattung die Beweislast eher bei dem liegen, der das Gegenteil behaupten wollte.

Nach dem bereits Gesagten dürften wir erwarten, dass sich bei Galen ein deutlich größerer Umfang an Geschmacksangaben findet als bei Dioskurides. Von den drei Büchern (VI–VIII), die sich thematisch (von der alphabetischen Anordnung abgesehen) mit den untersuchten Büchern des Dioskurides gut vergleichen lassen, finden sich Geschmacksangaben in 287 der insgesamt 474 Kapitel, also in 60 Prozent der Kapitel. Die Quote ist zwar um immerhin 10 Prozentpunkte höher als bei Dioskurides, doch noch nicht so hoch, wie angesichts des in Buch IV in Aussicht gestellten Forschungsprogramms zu erwarten wäre. Damit in Zusammenhang steht ferner eine Beobachtung, die Harig in seiner Monografie zur Intensitätsbestimmung, also der Einordnung der Drogen nach ihrer in vier Grade abgestuften Wirkstärke, gemacht hat. Wie er (1974: 143–145) zeigen konnte, liegen numerische Angaben nur für ein Drittel des galenischen Arzneischatzes vor, weswegen man den speziellen Teil wohl als unvollendet bezeichnen muss.

Dennoch sagt die Quote allein wenig über die Arbeitsweise des Pergameners aus. Vergleicht man seine Ausführungen mit denen des Dioskurides, fällt nämlich auf, dass dieser nicht nur Angaben bietet, die bei jenem fehlen, sondern dass er mitunter auch zu anderen Einschätzungen oder differenzierteren Urteilen kommt. Dioskurides gibt beispielsweise den Geschmack der *agrōstis*-Wurzel allein als „stark süß“ (γλυκείας ἰσχυρῶς, Dsc. IV 31) an, während Galen bemerkt, dass die *agrōstis* eine essbare Wurzel habe, „die, immer wenn sie weich ist, einerseits wässrig-süß schmeckt, andererseits aber etwas Beißendes und leicht Herbsaures hat“ (ἄγρωστις ἐδώδιμον ἔχει τὴν ῥίζαν, ἔστ’ ἂν ᾖ μαλακή, γλυκεῖα μὲν ὑδατώδης, δριμὺ δέ τι καὶ ὑπόστρυφνον ὀλίγον ἔχουσα, XI 810.15–17 K.). Der schon oben als Beispiel angeführte Baumpilz *agarikon* habe laut Dioskurides zu Beginn einen süßlichen, nach dem Kauen aber einen bitteren Geschmack (Dsc. III 1). Galen erscheint er ebenfalls „beim ersten Kosten irgendwie süß, wenig später aber leicht bitter und mit der Zeit führt er den Eindruck einer gewissen beißenden Schärfe und geringen Adstringenz herbei“ (κατὰ μὲν τὴν πρώτην γεῦσιν γλυκεῖά τις, ὑπόπικρος δὲ ὀλίγον ὕστερον φαινομένη καί τινος ἐν τῷ χρόνῳ δριμύτητος ἔμφασιν ἐπάγουσα, καὶ βραχείας στύψεως, XI 813.12–15 K.). Der Detailreichtum dieser Beschreibung erscheint manchen Interpreten zwar befremdlich („rather obscure“, Totelin 2018: 62), doch belegt er eindrücklich, für wie wichtig Galen diesen Aspekt in der Pharmakologie offensichtlich erachtete und man darf wohl vermuten, dass er dieselbe Akribie bei allen 474 Pflanzenlemmata würde angewendet haben, wenn er die Möglichkeit dazu gehabt hätte. Aber auch ungeachtet der Tatsache, dass er seinen Katalog in dieser Hinsicht nicht vervollständigen konnte, sind die genannten Beispiele keine Einzelfälle und es lassen sich hier noch viele weitere Angaben finden, die über das von Dioskurides Mitgeteilte hinausgehen, seine Einschätzungen ergänzen oder modifizieren – und zwar allein bei den Vegetabilia in über 100 Fällen.^18^

Was bedeutet dies für die Arbeitsweise Galens? Offensichtlich konnte er sich für viele Angaben nicht allein auf seine Hauptquelle stützen, die in diesem Belang nicht immer differenziert genug, teilweise auch unpräzise oder gar fehlerhaft war. Konnte er also auf eine andere Quelle zurückgreifen? Von den Autoren, die laut Galen (XI 789–798 K.) über *Simplicia* geschrieben haben, kommen eigentlich nur Sextius Niger, vielleicht Krateuas oder Diokles infrage. Wir haben indes keinen Anlass zu glauben, dass Galen Geschmacksqualitäten aus Sextius übernahm. Weder sind dessen Angaben, sofern wir sie durch Vergleich mit Plinius erschließen konnten, ausführlicher oder differenzierter, noch bezieht sich Galen in den sieben Fällen, wo Plinius Geschmacksangaben aus Sextius schöpft, die bei Dioskurides fehlen, eindeutig auf den Text des Sextius. Über die anderen beiden Autoren lässt sich, wie oben skizziert, wenig sagen. Es sollte aber zu denken geben, dass in Galens Zitaten aus den Werken dieser Autoren Geschmacksqualitäten keine Erwähnung finden. Hätte er denn überhaupt die *Materia medica* des Dioskurides als die „vollendetste“ (τελεώτατα, XI 794.11 K.) Darstellung ihrer Art loben können, wenn die anderen Autoren umfangreichere oder präzisere Angaben boten? Die Antwort mag sich jeder selbst geben. Wie die Dinge liegen, kommt vornehmlich eine Option in Betracht: Galen hat die abweichenden oder über Dioskurides hinausgehenden Geschmacksangaben selbst erhoben. Dass diese Vermutung nicht abwegig ist, dürfte aus den vielen Bemerkungen Galens, wo dieser um botanische und pharmazeutische Autopsie wirbt, eigentlich selbstverständlich sein. Dass Galen, dem als Leibarzt des Kaisers die wertvollen Magazine mit Arzneidrogen aus dem ganzen Reich offenstanden und der zudem viele Reisen unternommen hat, die allein der Beschaffung von Arzneimitteln dienten, dass also dieser Arzt wohl mehr als jeder andere Zeitgenosse auch die Möglichkeiten zum genauen Studium der Heilmittel hatte, wird wohl niemand bezweifeln.^19^

Da es nun plausibel erscheint, dass ein Großteil der protokollierten Geschmacksangaben tatsächlich auf das Konto Galens gehen,^20^ können wir als nächstes die eigentliche Tätigkeit des Schmeckens genauer untersuchen, und zwar unter der Bedingung der Möglichkeit, dass es sich um galenische Forschung handelt. Hier sind wir natürlich an das gebunden, was uns der Zufall der Überlieferung als Quellenmaterial zur Verfügung stellt. Die Ausgangslage ist jedoch nicht so hoffnungslos, wie ein Neuzeithistoriker annehmen könnte, der es gewohnt ist, sein Material aus so vielfältigen Quellen wie Labortagebüchern, unpublizierten Forschungsdaten, Korrespondenzen etc. speisen zu können. Denn auch das macht die Faszination des Autors Galen aus, dass sein gewaltiges Œuvre von biografischen Anmerkungen durchzogen ist und unsere Hauptquelle, die Heilpflanzenkataloge der Bücher VI bis VIII, weniger einen durchkomponierten und abgeschlossenen Traktat darstellen als vielmehr ein verschriftlichtes *work in progress*. Die folgenden Angaben sind für das Verständnis der pharmakologischen Forschung Galens daher sehr erhellend, wobei nicht verschwiegen werden darf, dass uns eine unabhängige Quelle zur Verifikation fehlt:

Galen bezog die Heilpflanzen entweder von vertrauensvollen Kontaktpersonen „teils aus Groß-Syrien, Palästina, Ägypten, Kappadokien, andere aus Pontus, ebenso aus Makedonien und den Ländern im Westen, wo Kelten, Iberer und in dem gegenüberliegenden Lande Mauretanier wohnen“ (*De antidotis* XIV 8.14–9.2 K.) oder bei in Rom ansässigen, aber oft weniger vertrauensvollen Händlern (Fälschungen waren sehr verbreitet, vgl. XIV 7.6 K.). Außerdem konnte Galen als Leibarzt unter anderem Mark Aurels (reg. 161–180) auf die kaiserlichen Magazine zurückgreifen, die mit wertvollen Spezereien insbesondere aus Kreta, dem „botanical Eden“ (Nutton 2013: 252), gefüllt waren. Von dort kamen alljährlich im Sommer die Kräuter der kaiserlichen Sammler in großen Körben an und wurden von Drogenhändlern unter anderem anhand des Geschmacks in ihrer Qualität beurteilt (*De antidotis* XIV 9–11). In Rom verfügte Galen entsprechend über einen der größten Arzneimittelvorräte seiner Zeit (Boudon-Millot 2012: 305, Anm. 6). Eine dritte Möglichkeit bestand darin, die Heilmittel selbst zu sammeln. So solle ein Arzt, wenn er sich gerade auf dem Land aufhalte und seine Medikamente nicht bei sich führe, „fähig sein, alles zu finden, was er zur Therapie benötige an Blüten, Früchten, Wurzeln, Rinden, Milchsäften, Blättern und Presssäften, Bäumen usw.“ (*Opt. med. cogn*. 12.3: CMG Suppl. Or. IV 125). In Latium unternahm Galen zu diesem Zweck botanische Exkursionen – im Wissen um die natürlichen Wuchsorte und die besten Erntezeitpunkte vieler Heilpflanzen in der Umgebung Roms (*De antidotis* XIV 30f. K.). Aber auch von den Pharmaka, die bei Händlern zu erwerben waren, solle man sich möglichst eine Kenntnis ihrer Herkunft – das heißt bei Arzneipflanzen ihres natürlichen Standorts, bei mineralischen Heilmitteln ihrer Lagerstätten – verschaffen (*De antidotis* XIV 7–9 K.). Diese Studien haben möglichst unter Anleitung eines erfahrenen Lehrers zu erfolgen; keinesfalls hinreichend sei ein bloßes Bücherstudium und das Betrachten von Pflanzenabbildungen. Auf keinen Fall solle man es nämlich „den Kapitänen gleichtun, die die Steuermannskunst nur aus Büchern erlernten“ (XI 797.1f. K.). Es sei hierzu viel Erfahrung nötig und es „genügt nicht, die Begutachtung (διάγνωσις) der Ingredienzien der Heilmittel einmalig, zweimalig oder dreimalig besorgt zu haben, sondern sehr viele Male“ (*De antidotis* XIV 6.14f. K.). Wenn man keine persönliche Erfahrung mit einem Arzneimittel habe, so solle man dies auch offen bekennen (*De san. tu.* VI 196.10–14 K.) – eine Maxime, der Galen selbst beispielsweise im Zusammenhang mit der im dritten Buch bei Dioskurides behandelten Pflanze *asklēpias* vorbildlich nachkommt (ΧΙ 840.11 Κ.).

Diese Informationen mögen genügen, um eine Vorstellung von Galens Umfeld und seinen Arbeitsmöglichkeiten zu erhalten. Wie er bei der Prüfung der Heilmittel konkret vorgeht, lässt sich mehreren Passagen aus Buch IV entnehmen. Besonders deutlich wird er in Kapitel 4 und 7 (XI 632f. und 642 K., siehe hierzu auch Harig 1973: 81–85 und Singer 2022: 183f.): Die Prüfung der Pharmaka geschieht nicht beliebig, sondern bedarf einer genauen Vorbereitung. Zunächst muss die Testperson gesund und in bestem körperlichem Zustand (XI 641.15f. K.) sein. Die eigentliche Geschmacksprüfung muss vorher – oder überhaupt immer wieder – sorgfältig eingeübt werden (γυμνάζειν ἐπιμελῶς, XI 632.7f. K.). Dazu beginnt man mit Substanzen, die nur eine einzige Qualität aufweisen (632.9 K., vgl. 643.2 K.: μιᾶς μόνης μετέχει ποιότητος) – etwa Zwiebeln oder Knoblauch (632.10 K.), die nur die beißend-scharfe Qualität besitzen. Diese kostet man kontinuierlich (συνεχῶς), zerkaut sie dabei so gründlich wie möglich (ἐπὶ πλεῖστον bzw. ἐπιμελῶς Par. gr. 2279) und versucht, sich den Geschmackseindruck exakt (ἀκριβῶς) einzuprägen (632.11–13 K.). Danach führt man dasselbe Prozedere mit bloß adstringierenden Drogen wie Galläpfeln und Gerbersumach (ἐπὶ κηκῖδος [κικίδος Kühn] τε καὶ ῥοῦ), dann mit bitteren und süßen Substanzen durch. Als Vergleichsmaß (μέσον) für den qualitätslosen (ἄποιον) Geschmack wird Wasser empfohlen (632.16f. K.). Wer in dieser Weise möglichst reine Geschmackseindrücke memoriert hat, kann sich an Drogen mit komplexen Qualitäten versuchen.

Auch wenn wir nicht wissen, wie Galen bei der eigentlichen Testphase der *Simplicia* im Detail vorging, wie viele Drogen er in einem Durchgang untersuchte, ob er vielleicht personelle Unterstützung hatte und sich über die Eindrücke austauschte, so darf zumindest vermutet werden, dass er sich zu den (mehrfach) untersuchten, also auch „geschmeckten“ Heilmitteln Aufzeichnungen machte und diese vielleicht geordnet nach Herkunft (Vegetabilia, Mineralia, Animalia) und nach den gängigen Bezeichnungen (halb-)alphabetisch sortiert in einer Art Kartei ablegte, aus der später das Konzept für den Katalog erstellt wurde. Man könnte auch vermuten, dass er aus dem Gedächtnis und seiner täglichen Praxis heraus seine Erfahrungen als Marginalien zur Hauptquelle hinzufügte, doch war die *Materia medica* von Dioskurides ja nicht alphabetisch gegliedert, weswegen eine irgendwie geartete vorherige Disponierung des Materials sicherlich zu erfolgen hatte. Seine Bemerkungen zum *agarikon* lesen sich ferner, als habe er die Geschmacksbeschreibung unmittelbar während oder kurz nach der Durchführung notiert – gewissermaßen noch mit dem Geschmack auf der Zunge und jedenfalls nicht aus dem Gedächtnis. Oder wäre es wahrscheinlich, dass ihm beim Abfassen des Kapitels über den Lorbeer zufällig einfällt, dass die „Wurzelrinde zwar weniger beißend und warm, dafür aber bitterer [ist] und auch eine bestimmte Adstringenz [besitzt]“ (XI 863.3–5 K.)? Ist es denkbar, dass ihm, beim Buchstaben Iota angelangt, wieder in den Sinn kommt, dass die Farnpflanze ἵππουρις/*hippouris* „eine mit Bitterkeit verbundene adstringierende Qualität“ (XI 889f. K.) aufweist? Oder dass ein anderes Farngewächs δρυοπτερίς/*dryopteris* „von süßer, beißender, leicht bitterer, bezüglich der Wurzel auch von herb-saurer Qualität“ (XI 865 K.), die Wurzel von ἐρυθρόδανον/*erythrodanon* „herb-sauer und bitter“ (XI 878 K.) ist, oder der Sadebaum βράθυ/*brathy* „Anteil an einer beißenden Qualität, ferner auch an einer Bitterkeit und Adstringenz“ (XI 853f. K.) hat? In all den genannten Fällen bietet Dioskurides weniger Informationen, und wenn man nicht voraussetzen möchte, dass Galen sich die entsprechenden Drogen vorher alphabetisch sortiert bereitgelegt hat, um den Katalog von Alpha bis Omega abfassen zu können, wird die Existenz einer Art Zettelkasten für Protokollnotizen äußerst wahrscheinlich.

Ein weiteres Beispiel seines methodischen Vorgehens ist die detaillierte Analyse der Zitronatzitrone (XII 77 K.). Hinsichtlich des Geschmacks nimmt er eine dreifache Gliederung der Fruchtwand vor, die sich ja auch im Längsschnitt schon makroskopisch aufdrängt (vgl. den Kommentar und die Zeichnung von *Citrus medica* L. bei Haars 2018: 308f.). So sei im Bereich des Samens die stechend-saure Qualität und trocknende Wirkpotenz vorherrschend, in der äußeren Rinde aber die beißende, während das essbare Fleisch zäh und schleimig sei, der Samen selbst aber bitter. Spätestens in diesem Kontext stellt sich die Frage, wie zutreffend seine Schilderungen sind, insbesondere wenn er von Dioskurides abweicht. Ein Vergleich mit modernen Angaben liegt allerdings nicht im Rahmen dieser Untersuchung. Es muss genügen zu bemerken, dass ein Vergleich da, wo er methodisch möglich ist,^21^ die galenischen Angaben in der Regel bestätigt (Haars 2018: 466f.). Es wäre angesichts der starken Rezeption, die diese Art der Pharmakologie erfahren hat, auch erstaunlich, wenn es sich anders verhielte.

Doch wenden wir uns noch einmal seiner Methode zu, die vielleicht am Beispiel der Zitronatzitrone am besten zum Ausdruck kommt: Zunächst werden verschiedene Pflanzenteile unterschieden und diese dann separat hinsichtlich ihres Geschmacks untersucht. Bis hierhin bewegt Galen sich durchaus im Rahmen der traditionellen, deskriptiven Pharmakognosie, auch wenn er vielleicht noch mehr an Details interessiert ist, als wir das bei Dioskurides (d. h. Sextius Niger) gesehen haben. Doch damit nicht genug: Nach Feststellung des Geschmacks, der „stechend-sauren Qualität“ des inneren Fruchtteils bemerkt Galen auch ein „trocknendes Vermögen“ und folgert aus beidem, „dass sie zum dritten Grad sowohl von den trocknenden als auch kühlenden Mitteln gehört“ (ὡς τῆς τρίτης εἶναι τάξεως ἀπὸ τῶν ξηραινόντων τε καὶ ψυχόντων, XII 77.6f. K.). Diese Folgerung hat nun nicht mehr den Charakter einer Protokollnotiz und lässt sich weder inhaltlich noch formal hinsichtlich der Verknüpfung in der vorhergehenden pharmazeutischen Literatur finden. Um zu verstehen, welche Bedeutung die Geschmackswahrnehmung für den pharmakologischen Wissenserwerb für Galen besessen hat, müssen wir im nächsten Teil also auf ebenjene Beziehung genauer eingehen.

## Geschmacksurteile als pharmakologische Indikatoren, Verifikatoren und Explananda

Neben der quantitativen Zunahme, Präzisierung und Korrektur von Geschmacksangaben lässt sich bei Galen auch eine Veränderung der Kontextualisierung dieser Angaben beobachten. Nur in wenigen Fällen stehen diese nämlich wie in der vorhergehenden pharmakognostischen Literatur isoliert da (z. B. XII 52.2f. K.). Meist sind sie eingebettet in eine wohldurchdachte Argumentation, die letztlich den Aufweis der durch die Elementarqualitäten und ihr Mischungsverhältnis hervorgerufenen pharmakologischen Effekte zum Ziel hat. Hierfür seien einige Beispiele genannt: „Die Wurzelrinde der Kaper hat vorherrschend eine bittere Qualität, als zweites eine beißende und danach eine saure. Damit ist klar, dass sie aus verschiedenen und streitenden Wirkungspotenzialen zusammengesetzt ist.“^22^ Dioskurides (II 173.2) bietet übrigens keine Geschmacksbeschreibung dieser Droge. Für Galen ist der Geschmack aber nicht nur Indikator für komplexe Zusammensetzungen und Mischungen der Elementarqualitäten, sondern kann auch zur Bestimmung ihrer Intensität herangezogen werden: „Das kleine *polion* ist nämlich auch beißender und bitterer als das große, sodass es aus dem dritten Grad der trocknenden und dem ausgefüllten zweiten Grad der wärmenden Mittel ist“^23^. Die Beobachtung, dass sich Unterschiede von wilden und kultivierten Pflanzen auch im Geschmack manifestieren, wurde auch von Dioskurides öfters gemacht (u. a. Dsc. III 45, siehe oben). Dass die Ursache für den oft kräftigeren Geschmack aber die höhere Intensität der Elementarqualitäten ist, findet sich erst bei Galen: „Die wilde Kichererbse ist in allen Belangen stärker als die kultivierte, deshalb ist sie wärmender und trocknender in dem Maße, wie sie auch beißender und bitterer ist.“^24^ Öfter noch als das Verhältnis zu den ersten Qualitäten wird der Zusammenhang mit den Arzneimittelwirkungen beleuchtet. So sei der Meerfenchel „irgendwie salzig im Geschmack und zugleich mit einer geringen Bitterkeit, und deshalb ist sein Wirkpotenzial zugleich reinigend und trocknend“^25^. Die Pflanze *chamaidrys* hingegen habe „vorherrschend die bittere Qualität, sie ist aber auch irgendwie beißend. Aus diesem Grund offensichtlich erweicht sie die Milz regelrecht und treibt den Urin und die Monatsblutung.“^26^*Sison* sei „warm und leicht bitter im Geschmack und deshalb harntreibend“^27^ und der Pfirsichbaum habe „in den Trieben und Blättern vorherrschend eine bittere Qualität und aus diesem Grund töten seine zerriebenen und um den Bauchnabel herum gelegten Blätter die Eingeweidewürmer“.^28^ Von der Ulme wird berichtet, dass „die Rinde in höherem Grade bitter und adstringierend“ ist, „sodass sie auch den Aussatz heilt“.^29^

Es ließen sich noch beliebig viele weitere Beispiele anführen, doch ist die Eigentümlichkeit der galenischen Wissenschaftsprosa hiermit wohl bereits hinlänglich deutlich geworden. Geschmacksangaben sind stets in größere Aussagezusammenhänge durch ätiologische Begründungen oder logische Folgerungen integriert. Es werden kausale Verbindungen hergestellt zwischen den Aussagen, dass Droge x eine bestimmte Geschmacksqualität – im Folgenden G(x) – oder eine bestimmte Elementarqualität oder -wirkung E(x) zukommt, die zuweilen auch hinsichtlich ihrer Intensität I_E_(x) bestimmt wird, und den speziellen medizinischen Effekten oder partikularen Wirkungen *P*(x) der Heilpflanzen. Galen schöpft hierbei die vielfältigen Möglichkeiten der griechischen Sprache voll aus, um seine Argumentation wissenschaftlich zu fundieren. Da der Sprachverwendung Galens im Hinblick auf die epistemologische Bewertung der Geschmacksangaben eine große Bedeutung zukommt, soll diese im Folgenden für die Bücher VI bis VIII genauer untersucht werden. Hierzu wurden die Formulierungen zunächst nach ihrer Aussageabsicht eingeteilt, was allerdings in einigen Fällen reine Interpretationsfrage ist. Die folgende Gliederung versteht sich daher nur als Annäherung und Überblick mit Fokus auf der Funktion von Geschmacksurteilen und nicht als eine Untersuchung zur Argumentation Galens insgesamt. Als systematisch vorrangig ist die Verhältnisbestimmung G(x) zu E(x) in den Blick zu nehmen, die nach unserer Zählung in mindestens 48 Fällen vorliegt. Es lassen sich grundsätzlich folgende Aspekte feststellen:

1. 33 × E(x) unbekannt; aus G(x) als Indikator wird auf E(x) zurückgeschlossen. Die Korrelation wird ausgedrückt durch:
  (*aus G[x] wird I_E_ [x] bestimmt. Die Proportionalität wird ausgedrückt durch:)
  - 9× kausale Adverbien wie διὰ τοῦτο „aus diesem Grund“ (XI 864.9 K., 889.9 K., vgl. auch XII 20.18f. K., XII 58.9 K., 114.15 K., 156.15 K.*), διὸ καὶ (XI 841.12 K.*, XII 44.7 K., 60.5 K.*, 77.9 K.*)
  - 7× Evidenz anzeigende Ausdrücke wie ἐξ ὧν δῆλον ὡς „daraus erhellt, dass“ (XI 810.18 K., 834.11 K., 879.13 K.) oder sogar ἐξ ὧν ἁπάντων εὔδηλον (XI 813.15f. K.), ὅθεν δῆλον ὡς (XI 820.1 K.), ἐξ ὧν ἁπάντων δῆλον ὡς (XI 821.8 K.), καὶ δηλονότι (XI 851.16 K.)
  - 7× quantifizierende Vergleiche wie εἰς ὅσον καὶ „in dem Maße wie“ (XI 866.8 K.*), τοσούτῳ … ὅσον καὶ „in einem solchen Maße … wie auch“ (XI 868.4 K.*) oder εἰς ὅσον … εἰς τοσοῦτον (XI 686.12f. K.*, 869.5 K.*, XII 24.14f. K.*, 123.18f. K.), ὅσον περ (XI 877.8 K.*)
  - 6× konsekutive Konjunktionen wie ὥστε καὶ „sodass auch …“ (XI 833.13 K.*, XII 47.15 K.) oder einfach ὡς (XII 122.14 K.*, 107.5 K.*, 107.15 K.*, 125.18 K.*)
  - 5× qualifizierende Vergleiche wie ὥσπερ τῇ γεύσει … οὕτως καὶ τῇ κράσει/„wie im Geschmack … so auch in der Mischung [der E]“ (XI 856.14f. K., vgl. XII 52.18 K., vgl. XII 99.5f. K., vgl. auch 112.7 K., vgl. 131.10 K.)
2. 10× Gegebene E(x) werden durch G(x) als Verifikatoren bestätigt. Die Kausalität wird ausgedrückt durch:
  - 8× Partikel γὰρ (XI 812.1 K., 856.11 K., 865.3 K., XII 36.8 K., 52.18 K., 70.10 K., 106.4 K., 128.18 K.)
  - 1× ὅτι καὶ „weil auch“ (XI 815.7 K.)
  - 1× konsekutive Konjunktion ὥστε (XII 14.17 K.)
3. 6× Beobachtete G(x) werden durch E(x) erklärt. G(x) als Explananda. Die Kausalität wird ausgedrückt durch:
  - 3× kausale Adverbien wie καὶ διὰ τοῦτο „und aus diesem Grund …“ (XII 69.14 K.), διὸ καὶ „weswegen auch …“ (XII 92.10, 118.12 K.)
  - 3× Präpositionale Wendungen: ἀπό + E im Genitiv in der Bedeutung „ausgehend von E …“, XII 130.16f. K., oder mit διά + E im Akk. in der Bedeutung „aufgrund der E …“, XII 134.4 K., 135.13 K.

Wie man sieht, geht es Galen überwiegend darum, in einem heuristischen, induktiven Verfahren aus gegebenen G(x) auf die sie hervorrufenden E(x) zu schließen, wobei den G(x) eine Indikatorfunktion zukommt (Fall 1). In den anderen beiden Fällen sind die E(x) bereits bekannt – etwa aus der Tradition oder durch ein vorangegangenes heuristisches Verfahren –, und die G(x) werden in einem nachträglichen deduktiven Verfahren eingeführt. Im zweiten Fall geht es also darum, die – wodurch auch immer – gegebenen E(x) durch festgestellte G(x) zu bestätigen, wobei ihre Funktion als Verifikatoren impliziert wird. Im dritten Fall geht es darum, die gegebenen G(x) mittels der E(x) zu erklären. Da Galen das induktive und deduktive Verfahren in den einzelnen Kapiteln nicht kombiniert, kommt seine Argumentation nicht sofort in den Verdacht, zirkulär zu sein. Für alle drei Muster hat Galen bestimmte Ausdrucksweisen, die er präferiert.

Die andere Gruppe von Aussagen, die betrachtet werden muss, betrifft die Relation der Geschmacksqualitäten zu den ebenfalls empirisch beobachteten und tradierten Arzneimittelwirkungen.

4. 29× aus G(x) wird *P*(x) gefolgert: Die Kausalität wird ausgedrückt durch:
  - 15× konsekutive Konjunktionen wie ὥστε καὶ „sodass auch …“ (XI 819.12 K., 851.1 K., 855.5 K., 886.18 K., XII 13.8 K., 41.16 K., 42.2 K., 52.11 K., 109.7 K., 131.6 K., 155.14 K.), oder einfach ὡς (XII 56.16 K., 93.18f. K., 97.18 K., 143.17 K.)
  - 8× folgernde Partikel „folglich, daher, aus diesem Grund“ wie τοιγαροῦν (XI 891.8 K., XII 102.13 K., 106.15 K., 121.3 K., 126.3 K.), τοίνυν (XI 888.14 K.), ἄρα (XI 859.3 K., 863.5 K.)
  - 6× Evidenz anzeigende Ausdrücke wie ᾧ καὶ δῆλον „daraus erhellt, dass …“ (XII 9.12f. K.), δῆλον ὅτι καὶ … usw. (XI 824.6 K., 879.13 K.), (ἐξ ὧν) δηλονότι (XII 77.16 K., 153.9 K.), εὐλογῶς (XII 84.15f. K.)
5. 71× Gegebene *P*(x) wird durch G(x) begründet. Die Kausalität wird ausgedrückt durch:
  - 44× kausale Adverbien wie ὅθεν „wodurch, weshalb“ (XI 878.9 K., 884.1 K., XII 9.1 K., 40.12 K., 41.9 K., 54.12 K., 69.2 K., 88.14 K., 103.2 K., 120.10 K., 136.2 K.), διὸ καὶ „weswegen auch …“ (XI 819.3 K., 826.15 K., 830.12 f. K., 858.4f. K., 865.17 K., 884.10 K., XII 43.14 K., 44.7 K., 89.5 K., 92.6 K., 96.11 K., 121.7 K., 123.15 K., 154.2 K., 157.18 K.), καὶ διὰ τοῦτο „und aus diesem Grund“ (XI 834.19f. K., 837.17 K., 845.5 K., 853.2f. K., 853.12 K., 861.6 K.,862.5 K., 864.9 K., 880.4 K., XII 49.10 K., 54.4 K., 55.8 K., 68.16 K., 76.11 K., 93.7f. K., 128.8 K., vgl. 131.6 K., 147.2 K.)
  - 14× präpositionale Wendung διά + G im Akk. „aufgrund“ (XI 877.2 K., 879.4 K., XII 11.14 K., 12.18 K., 41.2–5 K., 60.8 K., 64.13 K., 79.4 K., 80.12 K., 85.14 K., 113.13f. K., 127.6 K., 136.16 K., 142.7 K.)
  - 5× Partizipialkonstruktion mit kausalem Sinn, z. B. XI 856.7 K., 867.13 K., XII 57.1 f. K., 152.16 K., 153.15 K.
  - 4× Partikel γάρ „denn“ (XI 815.16 K., XII 23.17f. K., 68.6 K., 109.17 K.)
  - 3 × G im instrumentalen Dativ in der Bedeutung „durch (oder vermittels) G wirkt die Droge …“: XI 883.11f. K., XII 9.15f. K., 101.1 K.
  - 1× Konstruktion mit dem Relativadverb ᾗ „insofern“ (XI 859.6f. K.)
6. 10 × G(x) und *P*(x) korrelieren in einem nicht näher bestimmten kausalen Verhältnis. Die Korrelation wird ausgedrückt durch:
  - 7× (ὅμοιον) κατά τε τὴν γεῦσιν καὶ κατὰ τὴν ἐνέργειαν (auch δύναμιν), XI 890.17f. K., XII 15.5f. K., XII 19.10–18 K., vgl. auch XII 27.1f. K., 107.17f. K., 116.4 K., 155.1–3 K.
  - 2× korrelatives μέν – δέ: „einerseits … andererseits“, Bsp. καὶ γευομένη μέν … καὶ τοῖς ἔργοις δὲ, XI 888.5f. K., vgl. auch XII 93.10f. K.
  - 1× quantitativer Vergleich: εἰς ὅσον … εἰς τοσοῦτον (XII 91.17 K.)

Die Untersuchung der Verhältnisbestimmung G(x) zu *P*(x) ergibt zunächst – für uns unerwartet –, dass diese mit 110 Instanzen mehr als doppelt so häufig auftritt wie E(x) zu G(x). Dies ist insofern überraschend, als es ja Galen vornehmlich um den Aufweis der E(x) beziehungsweise I_E_ (x) geht und G und P nur indirekt über E zusammenhängen. Man könnte daher vermuten, dass Galen hier mit verkürzten Syllogismen operiert, indem er den Zusammenhang mit E impliziert, und dass er G nicht tatsächlich als Ursache für P ansieht (dies bedürfte einer eingehenden Untersuchung, die hier nicht geleistet werden kann). Der insgesamt häufigste Fall (5) ist, dass ähnlich dem deduktiven Verfahren von 2) oder 3) gegebene *P*(x) durch gegebene G(x) eine Begründung oder Bestätigung erfahren, was in den meisten Fällen (5a) durch kausale Adverbien beziehungsweise Konjunktionen geschieht. Auch hinter den Formulierungen von Fall (5) steht eine ähnliche Intention, indem aus einer gegebenen G(x) eine *P*(x) gefolgert wird. Das letzte Muster schließlich erfasst Aussagen, die G(x) und *P*(x) miteinander wechselseitig korrelieren, ohne dass die Abhängigkeit thematisiert wird.

Abgesehen von der ebenfalls häufig auftretenden Verhältnisbestimmung von E(x) zu *P*(x) unter Nichtbeachtung von G(x), die für unser Thema aber nicht unmittelbar von Bedeutung ist und daher hier nicht untersucht wurde, haben wir die wichtigsten G(x)-Argumentationsmuster in den Büchern VI bis VIII damit erfasst. Grundsätzlich zeigt sich, dass die Feststellung von Geschmacksqualitäten eine zentrale Stellung in Galens pharmakologischen Analysen einnimmt, insofern diese mit allen anderen Variablen in Verbindung gebracht werden und ihre Verknüpfung, wie wir gesehen haben, durch ganz unterschiedliche Arten von Junktoren auch wechselseitig geschieht. Die Geschmackswahrnehmung erfüllt dadurch nicht nur die Funktion, Arzneimittelwirkungen und ihre zugrunde liegenden Wirkungspotenziale anzuzeigen, sondern dient auch dazu, letztere zu bestätigen und liefert überhaupt auch den Anstoß für pharmakologische Erklärungsversuche. Insofern können wir sagen, dass die Geschmackswahrnehmung eine dreifache Funktion für den Erwerb von Wissen in der speziellen Pharmakologie Galens erfüllt: 1.) eine Indikatorfunktion, 2.) eine Kontroll- oder Verifikatorfunktion und 3.) eine Funktion als Lieferant erklärungsbedürftiger Phänomene (Explananda), die Galen zum Anlass ätiologischer Betrachtungen nimmt.

## Ein zukunftsweisender Ansatz?

In Friedrich August Flückigers (1828–1894) im Jahr 1867 erschienenem Lehrbuch *Pharmakognosie des Pflanzenreichs*, mit dem er die gleichnamige Disziplin nach herrschender Meinung auf eine wissenschaftliche Grundlage stellte, begegnet uns eine Einteilung, die uns eigentümlich bekannt vorkommt: Schon im Inhaltsverzeichnis werden Wurzeln und Rhizome klassifiziert in „aromatische, von schleimigem oder süßem Geschmacke, adstringierende, bitterliche oder bittere, von kratzendem oder scharf-brennendem Geschmacke“. Genauso geht er dann auch bei den Rinden, Blättern und Früchten vor – und stellt somit in einer Zeit, als chemische Methoden längst eine Art Deutungshoheit in der Pharmazie reklamiert hatten, „an die Sinnesorgane höchste Ansprüche“ (Haug 1985: 289).

Zwar mag die Einteilung aus pragmatischen Überlegungen erfolgt sein. Dennoch sollte es uns zu denken geben, dass in dieser Disziplin, der im 19. Jahrhundert ein ganz anderes Verständnis von Wissenschaft zugrunde liegt, eine Systematik maßgeblich auf Grundlage der Geschmackswahrnehmung entwickelt werden und paradigmatisch Eingang in ein Lehrbuch für Pharmazeut*innen finden konnte. Die Geschmacksprüfung der Vegetabilia ist, wie oben ausgeführt, noch immer ein Gegenstand des Pharmaziestudiums. Auch der Autor des vorliegenden Artikels hat auf diese Weise die Leistungsfähigkeit der gustatorischen Methode erfahren, was – und das soll nicht verschwiegen werden – sein Verständnis der galenischen Pharmakologie und ihrer wissenschaftsgeschichtlichen Bedeutung nicht unwesentlich beeinflusst hat. Obwohl Galens Argumentation, wie wir ansatzweise gesehen haben, unter den Verdacht fällt, zirkulär zu sein und aus heutiger Sicht auf vollkommen falschen Prämissen beruht, kann man ihr eine gewisse Überzeugungskraft nicht absprechen. Hierbei haben möglicherweise gerade die Geschmacksurteile einen großen Anteil, suggerieren sie doch dem/der Leser*in, dass man sich stets auf festem empirischem Boden bewegt. So mag auch die kühnste Hypothese akzeptiert werden, wenn die Argumentation mit der Feststellung abschließt, dass Absinth bitter schmeckt. Doch der starke Einbezug der Geschmackswahrnehmung ist viel mehr als nur ein rhetorischer Trick. Mit ihrer Hilfe gewinnt der Pharmakologe überhaupt erst ein vollständiges Bild, was die Wirkung eines Heilmittels ausmacht, und kann (innerhalb bestimmter Grenzen) zu einer Einteilung gelangen, die sich in der Therapie fruchtbar machen lässt (etwa durch die Einteilung Adstringentia – Bitterstoffdrogen – Kaustika).

Es könnte aufschlussreich sein zu untersuchen, ob das starke Eintreten für die Geschmacksprüfung der *Simplicia* bei Galen implizit oder explizit seinen Niederschlag in den drogenkundlichen Werken bis in die Neuzeit gefunden und wie Galens Lesern seine Lehre geschmeckt hat. Die Schwierigkeiten, die sich etwa durch die Übersetzung der Geschmacksqualitäten ergeben, sollten hierfür jedenfalls kein unüberwindbares Hindernis darstellen. Mit ihnen waren die Rezipienten des Galenismus im Lauf der Geschichte regelmäßig konfrontiert und haben sie durch unterschiedliche hermeneutische Anstrengungen zu bewältigen versucht. Dabei wurden die Pharmaka auch immer wieder neu geschmeckt und die Ergebnisse mit den antiken Autoren verglichen.^30^ Das pharmakologische System Galens bietet in seiner historischen Entwicklung somit tiefe Einblick in eine andere, sinnliche Forschungspraxis, in der nur Fortschritte macht, wer bereit ist, über Geschmack zu streiten.

## Footnotes

1 Hier wäre neben Harig (1973) außerdem zu nennen: van der Eijk (1997), Haars (2018: 70–75) und zuletzt Singer (2022), der das von Philip van der Eijk analysierte Konzept der „qualifizierten Erfahrung“ sehr überzeugend auch hinsichtlich der Verknüpfung von Primär- und Sekundärqualitäten untersucht.

2 In der Ausgabe von Kühn findet sich diese Schrift in den Bänden XI und XII unter dem längeren Titel περὶ κράσεως καὶ δυνάμεως τῶν ἁπλῶν φαρμάκων (*De simplicium medicamentorum temperamentis ac facultatibus* = *De simpl. med*.). Als authentisch dürfte nach den besseren griechischen Handschriften und den Selbstzitaten Galens aber der Titel περὶ τῆς τῶν ἁπλῶν φαρμάκων δυνάμεως gelten, siehe hierzu zuletzt Petit (2020: 95).

3 Zur Interpretation dieser Textstelle siehe auch Barnes (1997: 23).

4 Vgl. insbes. XI 450–452 K. Auf weitere Stellen gehen ein: Harig (1973: 70–75; dessen Übersetzung der Termini ich leicht abgewandelt übernommen habe) und Haars (2018: 71–75). Die Übersetzung von Geschmacksqualitäten ist in mehrfacher Hinsicht problematisch, wie Singer (2022: 179), Anm. 46 zu Recht herausstellt. Es seien hierbei insbesondere drei oft in Konflikt stehende Aspekte zu einem Kompromiss zu bringen: 1.) Idiomatik der Zielsprache, 2.) Herausforderung des antiken Autors, seinen Geschmackseindruck in Worte zu fassen, 3.) die bei Galen zugrunde liegenden physiologischen Vorstellungen, die stark von den unsrigen abweichen.

5 Wichtige Galenstellen hierzu sind *De simpl. med*. I 38f.: XI 449–455 K. und V 26: XI 785f. K. Weitere Stellen hat Harig (1973: 70–75) mit großer Sorgfalt zusammengetragen. Sie wurden in der Tabelle bei Haars (2018: 72f.) um weitere Angaben (insbesondere Beispiele) ergänzt.

6 Aus Platzgründen können hier nur die Stellen angegeben werden. Eine Synopse mit Textexzerpten und Übersetzungen kann im Forschungsdatenrepositorium der Universität Marburg abgerufen werden (siehe erste Anm.*). Dsc. III 3 – Pl. XXV 71 (e S. N.); Dsc. III 4 – Pl. XXV 95–98 (e S. N.); Dsc. III 9 – Pl. XXII 47 (e S. N.); Dsc. III 18 – Pl. XXVII 29 (e S. N.); Dsc. III 19 – PIin. XXII 40 (e S. N.); Dsc. III 20 – Pl. XIII 115; Dsc. III 34 – Pl. XX 147f. (e S. N.); Dsc. III 35 – Pl. XX 144 (e S. N.); Dsc. III 38 – Pl. XX 245f. (e S. N.); Dsc. III 40 – Pl. XXI 53 u. 151 (e S. N.); Dsc. III 42 – Pl. XII 111; Dsc. III 44 – Pl. XXI 29 u. 132 (e S. N.); Dsc. III 50 – Pl. XXV 32; Dsc. III 51 – Pl. XIX 165 u. XX 168. 187; Dsc. III 52 – Pl. XX 30f. (e S. N.); Dsc. III 53.1–2 – Pl. XX 36 (e S. N.); Dsc. III 57 – Pl. XIX 164; Dsc. III 59 – Pl. XX 159–162 (e S. N. cf. XIX 161); Dsc. III 60 – Pl. XX 159f. (e S. N.) cf. XIX 161; Dsc. III 65 – Pl. XIX 124 (e Theophr.), XX 117 (e S. N.); Dsc. III 66 – Pl. XX 118; Dsc. III 67 – Pl. XX 117 (e S. N.), XIX 162; Dsc. III 71 – Pl. XX 255 (aus Diokles, vgl. fr. 210 v. d. E.); Dsc. III 74 – Pl. XXIV 99 f. (e S. N.); Dsc. III 81 – Pl. XX 197 (e S. N.); Dsc. III 83 – Pl. XII 126; Dsc. III 84 – Pl. XII 107 (e S. N.); Dsc. III 98 – Pl. XXIV 130 (e S. N.); Dsc. III 105 – Pl. XX 241–244 (e S. N.); Dsc. III 106 – Pl. XXIV 136 (e S. N.) und Dsc. III 132 – Pl. XXV 75.

7 Dsc. III 48: σπέρμα εὐῶδες καὶ πυρωτικόν, ῥίζας […] παχὺν ἐχούσας τὸν φλοιόν, ὑπόπικρον ἐν τῇ γεύσει […] εἰσὶ δὲ τῶν ῥιζῶν βελτίους αἱ […] πυρώδεις ἐν τῇ γεύσει καὶ ἀρωματίζουσαι· […] τοῦ δὲ ὀποῦ διαφέρει ὁ πικρότερος τῇ γεύσει.

8 Dsc. III 80: διαφέρει δὲ αὐτοῦ [sc. τοῦ ὀποῦ] ὁ […] μὴ πρασίζων μηδὲ ἀπηνὴς πρὸς τὴν γεῦσιν […] ὁ μέντοι Κυρηναϊκός […] τῇ τε ὀσμῇ ἐστι προσηνέστατος, ὥστε γευσαμένῳ μηδὲ τὸ στόμα πνεῖν. Diese Angabe fehlt bei Pl. XIX 38–46 (e S. N.), vgl. PIin. XXII 100–106 (e S. N.).

9 Keine Parallele hat das πήγανον ἄγριον (Dsc. III 46), welches auch μῶλυ genannt wird und von dessen Samen gesagt wird, dass er für den Geschmack ziemlich bitter sei (σπέρμα […] πικρὸν ἱκανῶς πρὸς τὴν γεῦσιν). Ebenso die Hirschzunge, die einen sauren Geschmack habe (Dsc. III 107: γευσαμένῳ στρυφνή).

10 Beide betonen die Bitterkeit des kleinen Centauriums: Dsc. III. 7: ῥίζα […] γευομένῳ δὲ ἐμποιεῖ πικρίαν – *centaurium* […] *amaritudinem summam*, Pl. XXV 68 (e S. N.). Dsc. III 8 fand die Wurzel vom weißen Chamaileon mit einem schweren Duft leicht süßlich (ῥίζαν […] βαρύοσμον, γλυκεῖαν), so auch Plinius XXII 45 (e S. N.: *radice dulci, odore gravi*). Vgl. ferner Dsc. III 10 – Pl. XXVII 64 (e S. N.); Dsc. III 13 – Pl. XXIV 107 (e S. N.), Dsc. III 15 – Pl. XXVII 122 (e S. N.); Dsc. III 24 – Pl. XXI 160 (e S. N.; *amaritudo absinthii*, ist wohl etwas zu hoch gegriffen); Dsc. III 55 – Pl. XXVII 136 (e S. N.); Dsc. III 68 – Pl. XXVII 133 (e S. N.; vgl. XIX 187, wo gesagt wird, dass ihre Wurzel wie Myrrhe schmeckt: *zmyrnium* […] *murramque radice resipit*); Dsc. III 72 – Pl. XXV 110 (e S. N.); Dsc. III 78 – Pl. XXV 117 (e S. N.). Berücksichtigen wir eine von Wellmann zu wenig gewichtete Handschriftengruppe, stimmen beide auch hinsichtlich des nur beißenden Geschmacks der λευκάς überein; vgl. Dsc. III 99: ἡ ὀρεινὴ […] δριμύτερον καὶ μικρότερον ἔχουσα τὸν καρπὸν – ebenso auch Plinius: *leucas* […] *silvestrem* […] *semine acriore*, Pl. XXVII 102 (e S. N.). Marie Cronier (Paris) gab uns freundlicherweise folgenden Hinweis (brieflich am 14.02.22): „j’ai regardé tous les manuscrits importants qui contiennent ce chapitre (beaucoup de mss l’omettent): E (Scor. R.III.3) f. 55r, F (Laur. 74.23) ff. 120v–121r, G (Vat. gr. 284) f. 63r, P (Par. gr. 2179) f. 49v, W (Athos Meg. Laur. Ω 75) f. 190v. Parmi eux, seulement deux (P et F) donnent Λευκὰς ἡ ὀρεινὴ πλατυφυοτέρα τῆς ἡμέρου ἐστὶ καὶ δριμύτερον καὶ πικρότερον ἔχουσα τὸν καρπὸν καὶ ἀστοώτερον. L’une des traductions arabes est d’accord avec deux. Mais P, F et l’arabe forment une branche spécifique du stemma codicum et remontent à un ancêtre commun. Tous les autres manuscrits (E, G et W) donnent μικρότερον. Le latin n’est pas exploitable (lenae ou leue).“ Vgl. ferner: Dsc. III 124 – Pl. XXVII 62 (e S. N.); Dsc. III 128 – Pl. XXVI 97 (e S. N.); Dsc. III 130 – Pl. XXVII 121 (e S. N.); Dsc. III 133 – Pl. XXVII 25 (e S. N.); Dsc. III 136 – Pl. XXI 175 (e S. N.); Dsc. III 138 – PIin. XXI 176 (e S. N.); Dsc. III 140 – Pl. XXVII 84 (e S. N.); Dsc. III 152 – Pl. XXV 124 (e S. N.) und Dsc. III 157 – Pl. XXVI 86 (e S. N.).

11 Seine Quelle ist Thphr. HP IX 16.2: Τὸ δὲ ψευδοδίκταμνον […] χεῖρον δὲ πολλῷ καὶ ἀσθενέστερον. ἔστι δὲ εὐθὺς ἐν τῷ στόματι φανερὰ τοῦ δικτάμνου ἡ δύναμις· διαθερμαίνει γὰρ ἀπὸ μικροῦ σφόδρα.

12 Dsc. III 82: ἐκλέγου δὲ τὸν […] δριμύν. δυσδοκίμαστος δέ ἐστι τῇ γεύσει λαμβανόμενος διὰ τὸ ἅπαξ δηχθείσης τῆς γλώσσης ἐπιμένειν τὴν πύρωσιν ἐφ’ ἱκανὸν χρόνον, ὥστε πᾶν τὸ προσαγόμενον εὐφόρβιον δοκεῖν εἶναι.

13 Pl. XXV 79 (e S. N.): *etiam levi gustu os accensum diu detinens et magis ex intervallo*.

14 Beispielsweise nennt Dsc. III 2 die Rhabarberwurzel schleimig, Plinius dagegen „wärmend“; beide bemerken eine leichte Adstringenz und dass sie beim Kauen gelblich wird: ἔγγλισχρόν τε κατὰ τὴν γεῦσιν μετ’ ἀνειμένης στύψεως διαμασηθέν τε ἔνωχρον καὶ χροκίζον ποσῶς τῷ χρώματι, vgl. Pl. XXVII 128 (e S. N.): *calfaciens gustu et adstringens. eadem trita vini colorem reddit ad crocum inclinantem*. Vgl. ferner: Dsc. III 5 – Pl. XXII 24 (e S. N.). Dsc. III 6 – Pl. XXV 66 (e S. N.). Dsc. III 23 – Pl. XXVII 45 (e S. N.). Dsc. III 26 – Pl. XXVII 131 (e S. N.). Dsc. III 33 – Pl. XXII 146 (e Theophr., S. N.). Dsc. III 49 – Pl. XXV 30; Dsc. III 69 – Pl. XXII 79 (e S. N.); Dsc. III 85 – Pl. XXIV 128 (e S. N.); Dsc. III 111 – Pl. XXV 63 und Dsc. III 116 – Pl. XXVI 108.

15 In Bezug auf *selinon* (Dsc. III 64) zitiert Plinius Chrysippos aus Knidos (4./3. Jh. v. Chr.), der einen beißenden und brennenden Geschmack bezeugt: *Chrysippus feminam* [sc. *Apium*] *esse dicit* […] *sapore acri et fervido*, Pl. XX 112–113 (e S. N.). Vgl. ferner Pl. XX 255 (e S. N.) – Dsc. III 71; Pl. XXVII 83 (e S. N.) – Dsc. III 86; Pl. XXVII 32 (e S. N.) – Dsc. III 90; Pl. XXV 45 (e S. N.) – Dsc. III 97; Pl. XXVI 84 (e S. N.) – Dsc. III 151 und Pl. XXVI 85 (e S. N.) – Dsc. III 154.

16 Vgl. beispielsweise Dsc. III 4, der die Wurzelrinde der großen Aristolochia als aromatisch beschreibt (ἀριστολοχεία […] μακρά […] φλοιὸν ἐχούσας παχὺν καὶ ἀρωματίζοντα) – eine Angabe, die bei Krateuas fr. B 1 Wellm. fehlt. Auch in den anderen Fragmenten fehlen die von Dsc. genannten Geschmacksqualitäten regelmäßig. Eine Ausnahme sind die beiden Anemonenarten, wobei die „beißend-scharfe“ Eigenschaft als Wirkung und nicht als Qualität aufgefasst wird: Dsc. II 176: ἀνεμώνη […] δύναμιν δὲ ἔχουσι δριμεῖαν ἀμφότεραι – vgl. Krateuas fr. B 4 Wellm.: δύναμιν ἔχει δριμεῖαν, weswegen unsere oben gemachte Aussage nicht eingeschränkt werden muss.

17 Dass Theophrast Geschmacksangaben nicht systematisch erhoben hat, bedarf keiner näheren Untersuchung. Wie die theophrastische Geschmackslehre seiner Botanik mit derjenigen Galens zusammenhängt, bedüfte hingegen einer eigenen Studie. In unserem Zusammenhang ist allein die Feststellung wichtig, dass Galen den Theophrast für Geschmacksangaben in seiner speziellen Pharmakologie nicht herangezogen hat. Man vergleiche die wenigen gustatorischen Notizen im neunten Buch, beispielsweise Thphr. HP IX 1.4: „Der [Saft] vom Silphium ist beißend“ mit Galen XII 123 K. (Geschmacksangabe fehlt); oder 2.5: „Das [Harz] der Kiefer vom Ida ist süßer“ (keine Parallele bei Galen); oder 4.7: „Das Blatt der Myrrhe schmeckt beim Kauen nach Terebinthe“ (die *folium*-Droge von σμύρνα ist bei Galen XII 127 K. nicht berücksichtigt); oder 4.10 zu XII 127 K.; oder 5.3: die Würmer gehen nicht in das Holz der Kassia wegen der Bitterkeit – bei Galen XII 13.7 K. ist sie hingegen „beißend-scharf und etwas adstringierend“; oder 11.2: das asklepiadische Panakes hat eine dicke salzige Rinde und 11.3: das Panakes des Herakles hat eine bitterliche Wurzel – davon kein Wort bei Galen XII 94f. K.; oder 13.1 – dazu XII 86f. K.; oder 13.2 Süßholzwurzel – zufällig übereinstimmend mit XI 858 K.; oder 13.4 (vgl. 20.4) die Osterluzei-Wurzel ist bitter – zufällig übereinstimmend mit XI 835.16 K. (hier aber zusätzlich beißend-scharf); oder 16.2 – dazu XI 863 K.; oder 18.1 – dazu XII 66f. K.; oder 20.3 – dazu XI 826 K. und 20.5 die Wurzel der *pteris* ist „süßsauer“ (γλυκύστρυφνος) – anders dagegen XII 109f. K.

18 Die Stellen sind gesammelt bei Haars (2018: 466, Anm. 4) und in den jeweiligen Einzelkommentaren besprochen. Siehe auch die Synopse mit Übersetzungen in den Forschungsdaten zu diesem Aufsatz.

19 Zu sämtlichen biografischen Aspekten, die Einfluss auf Galens pharmakologische Kenntnisse hatten, siehe ausführlicher Haars (2018: 6–10) mit weiterführender Literatur.

20 Aus der unpersönlichen Formulierung der Geschmacksangaben und der Abwesenheit des „Ich des Wissenschaftlers“ (Asper 2005: 23f. „je scientifique“), das sonst bei Galen nicht selten vorkommt, darf jedenfalls nicht geschlossen werden, dass Galen nicht der Urheber dieser Angaben ist.

21 Einige Schwierigkeiten sind bereits in Anm. 4 formuliert. Man kann ihnen teilweise entgehen, indem eindeutige Fälle gewählt werden. So ist es aus naturwissenschaftlicher und pragmatischer Sicht unerheblich, dass Galen vor 1800 Jahren gelebt hat, wenn er den Geschmackseindruck von Gerbersumach als „adstringierend“ oder „herb“ angibt. Dass diese Aussage plausibel ist, ergibt sich übrigens auch textimmanent, da Galen zusätzlich die gerbenden Eigenschaften anführt (XII 115f. K.).

22 Gal. XII 9 K.: Καππάρεως ὁ μὲν τῆς ῥίζης φλοιὸς ἐπικρατοῦσαν ἔχει τὴν πικρὰν ποιότητα, δευτέραν δὲ τὴν δριμεῖαν, ἐφεξῆς δὲ ταύτῃ τὴν στρυφνήν. ᾧ καὶ δῆλον ὡς ἐκ διαφόρων τε καὶ μαχομένων σύγκειται δυνάμεων.

23 Gal. XII 107 K.: καὶ γὰρ καὶ δριμύτερον καὶ πικρότερόν ἐστι τοῦτο [sc. Πόλιον τὸ μικρὸν] τοῦ μείζονος, ὡς ἐκ μὲν τῆς τρίτης τάξεως εἶναι τῶν ξηραινόντων, ἐκ δὲ τῆς δευτέρας συμπεπληρωμένης τῶν θερμαινόντων.

24 Gal. XI 877 K.: Ἐρέβινθος ἄγριος ἰσχυρότερος εἰς πάντα τοῦ ἡμέρου, τοῦτ’ ἔστι θερμότερός τε καὶ ξηραντικώτερος, ὅσον περ καὶ δριμύτερός τέ ἐστι καὶ πικρότερος.

25 Gal. XII 40 K.: Κρῆθμον ἁλμυρόν πώς ἐστι γευομένοις, ἅμα βραχείᾳ πικρότητι, διὸ καὶ ἡ δύναμις αὐτοῦ ῥυπτική τε ἅμα καὶ ξηραντική.

26 Gal. XII 153 K.: Χαμαίδρυς ἐπικρατοῦσαν ἔχει τὴν πικρὰν ποιότητα, ἔστι δὲ καὶ δριμεῖά πως. ἐξ ὧν δηλονότι δεόντως σπλῆνα τήκει καὶ οὖρα καὶ καταμήνια κινεῖ.

27 Gal. XII 123 K.: Σίσων θερμὸς καὶ ὑπόπικρός ἐστι τὴν γεῦσιν. ὅθεν οὐρητικός τ’ ἐστὶ […]

28 Gal. XII 76 K.: τὸ δένδρον ἔν τε τοῖς βλαστοῖς καὶ τοῖς φύλλοις ἐπικρατοῦσαν ἔχει τὴν πικρὰν ποιότητα καὶ διὰ τοῦτο αὐτοῦ τὰ φύλλα τὰς ἕλμινθας ἀποκτείνει λειωθέντα καὶ κατὰ τοῦ ὀμφαλοῦ ἐπιτεθέντα.

29 Gal. XII 109 K.: ὁ δὲ φλοιὸς [sc. Πτελέας] ἔτι καὶ μᾶλλον ὑπόπικρός τέ ἐστι καὶ στυπτικός, ὥστε καὶ λέπραν ἰᾶται.

30 Vgl. etwa die Kommentare des englischen Naturforschers William Turner (1509/10–1568) bei Teigen (1987: 62).
